# Treatment evaluation of Kami Guibi‐tang on participants with amnestic mild cognitive impairment using magnetic resonance imaging on brain metabolites, gamma‐aminobutyric acid, and cerebral blood flow

**DOI:** 10.1002/acm2.13443

**Published:** 2021-10-11

**Authors:** Seung‐Yeon Cho, Sharonkyuhee Kwon, Hee‐Yeon Shin, Ha‐Ri Kim, Jeong‐Hwa Kim, Soonchan Park, Chang‐Woo Ryu, Jung‐Mi Park, Richard A.E. Edden, Geon‐Ho Jahng

**Affiliations:** ^1^ Stroke and Neurological Disorders Center, Kyung Hee University Hospital at Gangdong, College of Korean Medicine Kyung Hee University Seoul Republic of Korea; ^2^ Department of Biomedical Engineering, Undergraduate School, College of Electronics and Information Kyung Hee University Yongin‐si Gyeonggi‐do Republic of Korea; ^3^ Department of Clinical Korean Medicine, Graduate School Kyung Hee University Seoul Republic of Korea; ^4^ Department of Radiology, Kyung Hee University Hospital at Gangdong, College of Medicine Kyung Hee University Seoul Republic of Korea; ^5^ Division of Neuroradiology, Department of Radiology The Johns Hopkins University School of Medicine Baltimore Maryland USA

**Keywords:** brain metabolite, cerebral blood flow, Kami Guibi‐tang (KGT), mild cognitive impairment

## Abstract

**Purpose:**

To evaluate the effectiveness of Kami Guibi‐tang (KGT) in the treatment of mild cognitive impairment (MCI) using magnetic resonance imaging (MRI) on brain metabolites, neurotransmitter, and cerebral blood flow (CBF).

**Methods:**

We randomly allocated a total of 30 MCI patients to a KGT (N = 16) or a placebo (N = 14) group and performed MRI scans before and after 24 weeks of treatment. The participants underwent brain magnetic resonance spectroscopy and MRI scans to obtain brain metabolites using Point‐RESolved Spectroscopy (PRESS) single‐voxel spectroscopy, gamma‐aminobutyric acid (GABA) neurotransmitter using Mescher–Garwood PRESS, and CBF using pseudocontinuous arterial spin labeling sequences using a 3.0 Tesla MRI system. We analyzed metabolite and neurotransmitter levels and CBF using repeated‐measure analysis of variance to evaluate between‐subject group effect, within‐subject treatment condition effect, and interaction of group by condition (group x condition).

**Results:**

The GABA+/creatine (Cr) ratio values were not significantly different between the before and after treatment conditions. The glutamate complex/Cr ratio difference before and after treatment was lower in the KGT group than in the placebo group, but was not statistically significant (*p* = 0.077). The result of region of interest–based CBF measurement showed that CBF values were significantly lower after treatment at Cluster 2 for the KGT group (*p* = 0.003) and the placebo group (*p* = 0.011), at hippocampus for the KGT group (*p* = 0.004) and the placebo group (*p* = 0.008), and at the fusiform gyrus for the KGT group (*p* = 0.002). Furthermore, the absolute CBF difference before and after treatment in the fusiform gyrus was significantly lower in the KGT group than in the placebo group (*p* = 0.024).

**Conclusions:**

Although a KGT treatment of 24 weeks showed some significant impact on the level of CBF, the Korean version of the mini‐mental state examination score was not significantly different between before and after treatment conditions, indicating that there was no memory function improvement after treatment in amnestic MCI patients. Therefore, further studies should be performed with a relatively larger population and extending the duration of the KGT treatment.

## 1 INTRODUCTION

Mild cognitive impairment (MCI) refers to the clinical state between normal aging and Alzheimer's disease (AD), where a person experiences a greater degree of memory loss than expected for age. Amnestic MCI (aMCI) is considered a clinically high‐risk condition for AD.^1^, ^2^ Therefore, it is important to prevent the progression to AD, but there are no recommended treatments for aMCI.^3^ Many researchers have tried to review the potential pharmacological treatment options, and clinical trials of potential treatments including herbal medicines are ongoing.^4^, ^5^

Kami Guibi‐tang (KGT) is a herbal medicine commonly used in Asia to treat forgetfulness, insomnia, or depression. Several experimental and clinical studies have investigated the effectiveness of KGT on cognitive impairment. Long‐term administration of KGT improved learning performance in the senescence‐accelerated mice model.^6^ KGT has been proven to significantly improve spatial memory impairment in mice.^7^ 5XFAD AD model mice treated with KGT significantly improved object recognition memory and decreased the number of amyloid plaques in the hippocampus and the frontal cortex.^8^ A clinical study of the effects of KGT on cognitive function in AD patients showed significant improvements in mini‐mental state examination (MMSE) scores after taking KGT for 3 months, but there was no significant change in cerebral blood flow (CBF) in single photon emission computed tomography (SPECT).^9^ In a crossover designed clinical trial to investigate the effect of KGT on cognitive function in patients with AD, MMSE scores significantly increased during KGT intake.^10^ However, clinical studies on the effectiveness of KGT in aMCI are insufficient, and there are few studies investigating the mechanism using magnetic resonance imaging (MRI).

MRI is often used to look at the effect of a treatment on biomarkers. Proton magnetic resonance spectroscopy (1H‐MRS) is a sensitive method to estimate alterations in the concentration of brain metabolites and neurotransmitters,^11^ including N‐acetylaspartate (NAA), creatine (Cr), myo‐Inositol (mI), choline (Cho), glutamate complex (Glx), and gamma‐aminobutyric acid (GABA).^12^, ^13^ Detecting changes in neuronal markers in the brain may be beneficial to assess treatment responses. Furthermore, the measurement of CBF changes using noninvasive arterial spin labeling MRI is another method to quantitatively evaluate the effectiveness of a treatment.^14^, ^15^, ^16^ A previous study has suggested that CBF is an appropriate measure to examine the neural response to pharmacological agents.^17^ Therefore, the objective of this study was to evaluate the effects of KGT treatment on the aMCI brain using MRI measures.

## 2 MATERIALS AND METHODS

### 2.1 Participants

The local institutional review board approved this cross‐sectional prospective study. This study was conducted between June 2017 and March 2019 at our institute hospital. We obtained informed consent from all participants. All the participants provided a detailed medical history and underwent neurologic examination, standard neuropsychological testing, and MRI scans. We assessed the participants’ cognitive functions using the Seoul Neuropsychological Screening Battery (SNSB),^18^ which is included in the Korean version of MMSE (K‐MMSE) and which measures the global cognitive ability. We included aMCI participants in this study based on the results of the neuropsychological examination and according to the Petersen criteria, as follows^1^, ^19^: (1) objective cognitive impairment as measured by SNSB with a Global Deterioration Scale of 3, a clinical dementia rating of 0.5, and a normal score on K‐MMSE; (2) no medication affecting cognitive functions at least 2 weeks before the study, including Gliatilin, Gliatamin, Ginexin, Tanamin, or other psychoactive drugs; (3) no medication change for underlying diseases at least 2 weeks before the study with no expected change in medication during the study period; and (4) age 55–90 years. The detailed study protocol was published elsewhere.^20^

We included a total of 30 participants with aMCI in this study and randomly allocated them to either the treatment or control group in a 1:1 ratio using the block randomization method with a block size of four. The participants, the assessor, the clinical trial pharmacist, and the researchers were blinded to the allocations throughout the course of the study. After randomization, the treatment group received KGT granules (3 g/pack) and the control group received placebo granules (3 g/pack) three times a day for 24 weeks. The KGT and control group included 16 and 14 participants, respectively. Table 1 summarizes the demographic characteristics of the participants.

**TABLE 1:** Summary of the statistical results of the demographic data and results of the neuropsychological tests obtained for the participants

Group	KGT		Placebo		RM‐ANOVA^a^		Group				
No. of participants	16		14		n/a						
Age (year)^b^	70.31 ± 7.77		70.14 ± 6.32		n/a		*p* = 0.949				
Gender (male/female)^c^	10/6 (M: 62.5%, *F*: 37.5%)		8/6 (M: 57.1%, *F*: 42.9%)		n/a		*p* = 0.352				
CDR (range)	0.5 (0–0.5)		0.5 (0–0.5)		n/a		n/a				

	Before(1a)	After(1b)	abs(Diff)(%)	Before(2a)	After(2b)	abs(Diff)(%)	BTS	WIS	G x Con	abs (Diff)^b^ p‐value
MMSE (/30)	27.69±1.89	27.94 ±1.65	4.60±4.56	26.29±2.05	26.71±2.13	6.75±5.60	*F* = 4.710 *p* = 0.039	*F* = 0.870 *p* = 0.358	*F* = 0.060 *p* = 0.808	0.257

*Note*: The abs(Diff) (%) is the absolute difference of the MMSE score between before and after treatments, divided by the MMSE value before treatment, and then multiplied by 100%. The abs(Diff) value was compared between the KGT and placebo groups by using the independent *t*‐test. The data of age and MMSE score are presented as the mean ± standard deviation. CDR scores are presented as the median (range) value.

Abbreviations: BTS, between‐subject group; CDR, clinical dementia rating; G x Con, group x condition; KGT, Kami Guibi‐tang; MMSE, mini‐mental state examination; RM‐ANOVA, repeated‐measure analysis of variance; WIS, within‐subject condition.

^a^ Result by RM‐ANOVA; The between‐subject effect was the participant's group of the 16 KGT and 14 placebo treatments. The condition for the within‐subject factor was those values before and after treatment. The test of within‐subject effect was evaluated for condition and group x condition interaction. G x Con is the group x condition interaction using Huynh–Feldt. The degree of freedom was 1.

^b^ *p* by independent *t*‐test.

^c^ chi‐squared test.

### 2.2 MRI acquisition

**FIGURE 1:**
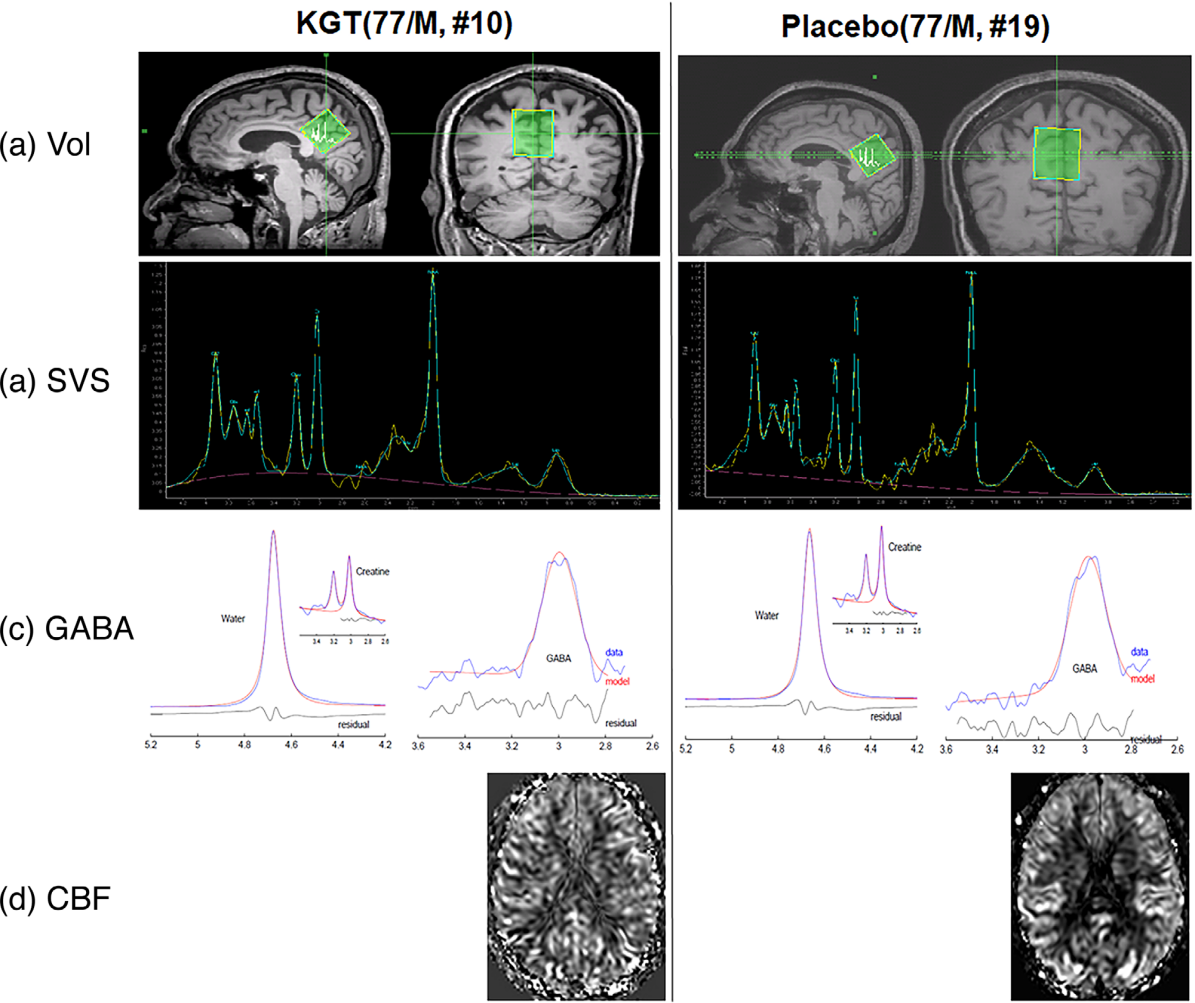
Representative magnetic resonance images and metabolite spectra; this figure shows the representative voxel of interest (a) for obtaining Point‐RESolved Spectroscopy (PRESS) single‐voxel spectroscopy (SVS) and the MEscher–GArwood (MEGA) PRESS spectra and the corresponding spectra for PRESS SVS (b) and MEGA PRESS (c), and spatially and cerebral blood flow maps (d) obtained before taking medicine from 77‐year‐old males among both KGT and placebo participants. In the PRESS SVS spectra (a), the yellow line indicates the raw data and the blue line the fitted spectrum of the raw data. In the MEGA PRESS spectra (c), the black color indicates the obtained raw data and the pink color indicates the fitted curves for water, creatine, and gamma‐aminobutyric acid (GABA)

The participants underwent brain MRIs at baseline and after 24 weeks of medication to obtain structural 3D T1‐weighted (3D T1W) image, Point‐RESolved Spectroscopy (PRESS) single‐voxel spectroscopy (SVS), MEscher–GArwood (MEGA) PRESS, and pseudocontinuous arterial spin labeling (pCASL) perfusion images with a 32‐channel sensitivity encoding coil using a 3.0 Tesla MRI system (Ingenia, Philips Medical System, Best, The Netherlands). Figure 1a depicts the representative volume of interest (VOI) for the proton PRESS SVS and MEGA PRESS. Table 2 summarizes brain MRI and MRS sequences, scan parameters, and the calculated quantitative indices obtained at baseline and after 24 weeks of medication using a 3.0 Tesla MRI system.

**TABLE 2:** Summary of brain magnetic resonance imaging (MRI) and magnetic resonance spectroscopy sequences, scan parameters, and quantitative indices obtained at baseline and after 24 weeks of medication using a 3.0 Tesla MRI system

Sequence name	Scan parameters	Calculated quantitative indices
Turbofield echo or MPRAGE structural 3D T1W	TR = 8.1 ms, TE = 3.7 ms, FA = 8°, FOV = 236 × 236 mm^2^, and voxel size = 1 × 1 × 1 mm^3^	
PRESS SVS	TR = 2 s, TE = 35 ms, FA = 90^o^, second order shimming, spectral resolution = 1.95 Hz, readout duration = 512 ms, sampling points = 1024, spectral bandwidth = 2000 Hz, water suppression technique = multiply optimized insensitive suppression train (MOIST) with 140 Hz window, averages = 128, phase cycles = 16, and scan duration = 5 min	NAA/Cr, Cho/Cr, mI/Cr, and Glx/Cr
MEGA PRESS	TR = 2 s, TE = 68 ms, FA = 90^o^, second order shimming, sampling points = 2048, spectral bandwidth = 2000 Hz, water suppression technique = MOIST with a 140 Hz window, BASING pulse = MPUWATSUP_INBASE_MEGA_EDIT with 15 ms pulse duration and 180 ^o^ flip angle, averages = 256, phase cycles = 16, ON/OFF = 1.9/7.46 ppm, and scan duration = 8 min 48 s	GABA/Cr
Single‐shot echo‐planar imaging (EPI) pCASL	Label duration = 1650 ms, postlabel delay = 1600 ms, label distance = 90 mm and 35 pairs of labeled control with two‐pulse background suppression, TR = 4 s, TE = 15 ms, FA = 90°, FOV = 230 × 230 mm, number of slices = 15, acquisition voxel size = 2.74 × 2.74 × 6 mm^3^, acquisition matrix = 84 × 84, reconstruction voxel size = 1.8 × 1.8 × 6 mm^3^, slice thickness = 6 mm with 1.2 mm, EPI factor = 37, and scan duration = 4 min 48 s	CBF

Abbreviations: 3D T1W, three‐dimensional T1‐weighted; CBF, cerebral blood flow; Cho/Cr, choline (Cho) divided by total creatine (Cr); FA, flip angle; FOV, field of view; GABA/Cr, gamma‐aminobutyric acid divided by Cr; Glx/Cr, glutamate complex divided by Cr (sum of 2.11 ppm through 2.45 ppm); MEGA, MEscher–GArwood; mI/Cr, myo‐Inositol divided by Cr (sum of 3.55 ppm through 3.63 ppm); MPRAGE, magnetization‐prepared rapid acquisition of the gradient echo sequence; NAA/Cr, N‐acetylaspartate divided by Cr; pCASL, pseudocontinuous arterial spin labeling perfusion images; PRESS, Point‐RESolved Spectroscopy; SVS, single‐voxel spectroscopy; TE, echo time; TR, repetition time.

First, for image registration and spatial normalization, a sagittal structural 3D T1W image was acquired with a turbofield echo sequence similar to the magnetization‐prepared rapid acquisition of the gradient echo sequence. Second, we acquired 1H‐MRS data using the PRESS SVS sequence to measure brain metabolite levels. The cubic voxel size of 30 × 30 × 30 mm^3^ was placed at the precuneus located in Brodmann area (BA) 7 and in the posterior cingulate (BA 23 and 31) area and extended inferiorly into the retrosplenial cortex (BA 29 and 30) of the brain. We defined the cubic voxel on the 3D T1W images on the midline sagittal plane and the coronal and sagittal reconstructed images. In this study, we did not put the data acquisition voxel in the hippocampus because it is usually challenging to obtain good MRS data due to field inhomogeneity and because it requires two separate MRS measurements for the right and left sides. Third, we ran the MEGA‐PRESS sequence obtained from Johns Hopkins University to quantify GABA. This sequence was based on the regular single‐voxel experiment, but with two added frequency‐selective MEGA editing pulses. The location and size of the cubic voxel size were the same as those in the SVS PRESS scan. Finally, we performed pCASL MRI using a single‐shot echo‐planar imaging (EPI) sequence for imaging acquisition mainly in the parietal and temporal lobes and in some cortex areas to quantify CBF in the brain. We also acquired a proton‐density image, called a reference image, using the same EPI sequence without the labeling part, to be used during the CBF mapping.

### 2.3 Postprocessing of MRI data

#### 2.3.1 Spectral quantification of PRESS SVS and MEGA‐PRESS data

We analyzed the acquired PRESS SVS data using version 7.0.1 of the MRSpectroView software (IntelliSpace Portal, Philips Medical Systems, Best, The Netherlands) with the following steps. First, we manually adjusted the phase of the spectrum to obtain a straight baseline and to adjust the fitted line to the acquired spectrum data. All the metabolite signals were fitted into the chemical shift range from 4.20 to 0.20 ppm. Second, we quantified the following metabolites: NAA at 2.01 ppm, Cho at 3.21 ppm, Cr at 3.03 ppm, mI at around 3.56 ppm, lipid at around 1.3 ppm, and Glx, which is a complex of glutamine and glutamate and one of the major excitatory neurotransmitters in the brain, between 2.2 ppm and 2.4 ppm. Third, we exported the metabolite levels into an Excel sheet to normalize the values by Cr level, which is a marker of membrane integrity, to minimize fitting errors for each spectrum. Figure 1b shows the representative PRESS SVS spectra obtained from the VOI for the 77‐year‐old males in the KGT (10th participant) and placebo (19th participant) groups. The yellow line indicates the raw data and the blue line, the fitted spectrum of the raw data.

We processed the acquired MEGA‐PRESS data using version 140709 of the Gannet software package provided by the Johns Hopkins University.^21^ Gannet consists of two main modules, GannetLoad and GannetFit. GannetLoad imports time‐domain data from a scanner and processes it into a frequency‐domain GABA‐edited spectrum and GannetFit uses nonlinear least‐squares fitting to integrate the edited GABA peak and Cr signal at 3 ppm and to produce GABA concentration estimates. We quantified GABA as a metabolite ratio, that is, the GABA+ signal in the difference spectrum relative to the total Cr signal in the OFF spectrum. Only the 3.0 ppm GABA+ and Cr signals were fitted. Measurements are denoted GABA+/Cr. Figure 1c shows the representative MEGA‐PRESS spectra obtained from the VOI of the 77‐year‐old males in the KGT (10th participant) and placebo (19th participant) groups. The black line indicates the raw data and the pink line, the fitted spectrum for water, Cr, and GABA. The fitting errors for the GABA spectra were 5.6% for water and 9.4% for Cr for the KGT participant and 5.1% for water and 7.9% for Cr for the placebo participant.

#### 2.3.2 pCASL images

**FIGURE 2:**
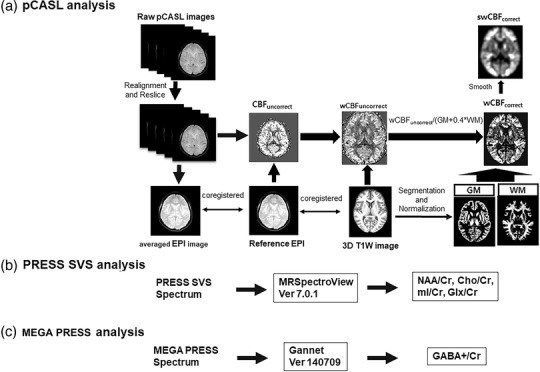
Diagram for data postprocessing steps; this figure shows the flow chart of the processing steps for pCASL (a), Point‐RESolved Spectroscopy (PRESS) single‐voxel spectroscopy (b), and Mescher–Garwood PRESS (c). The detailed explanations are also found in the part of the postprocessing of magnetic resonance imaging data in the Section 2

Figure 2 shows the diagram for data postprocessing steps. To obtain the CBF values, we performed the following steps using the Statistical Parametric Mapping Version 12 software (http://www.fil.ion.ucl.ac.uk/spm/software/spm12/). First, for the pCASL data, we realigned 35 pairs of control and the labeled EPI images of each subject to the first volume to obtain averaged EPI images and resliced them according to any motion. Then, we coregistered the proton‐density EPI, the reference image, and the created averaged EPI and mapped the voxel‐based CBF for each subject using a one‐compartment model embedded in a MATLAB code.^22^

Second, we coregistered the 3D T1W image and the reference EPI image and segmented the 3D T1W image into gray matter (GM), white matter (WM), and cerebrospinal fluid (CSF) using the CAT12 tool (http://www.neuro.uni‐jena.de/cat/) to obtain brain tissue volumes. The 3D T1W images were spatially normalized to the dementia template generated in our lab.^23^ The CBF map was therefore normalized into the dementia standard template using the deformation field information of the 3D T1W image.

Third, to correct the partial volume effect in the CBF maps at the voxel level, we corrected the CBF value in each voxel according to the following formula: CBF_correct_ = CBF_uncorrect_/(GM+0.4^*^WM), where CBF_correct_ and CBF_uncorrect_ are CBF values after and before partial volume correction, respectively. We assumed that the perfusion of WM is globally 40% of that of GM based on a previous positron emission tomography (PET) study.^24^ Additionally, we only included comparisons of CBF values in the voxels that had more than 50% of GM and WM (i.e., [GM+WM] > 0.5) to minimize the contribution of CSF and increase the statistical power. Finally, we performed a Gaussian smoothing using a full width at half maximum of 8 × 8 × 8 mm^3^ for the voxel‐based statistical analyses of CBF maps. Figure 1d shows the representative normalized CBF maps obtained from the 77‐year‐old males in the KGT (10th participant) and placebo (19th participant) groups.

### 2.4 Statistical analyses

#### 2.4.1 Demographic data and clinical outcome scores

To evaluate the difference between the KGT and placebo groups, we compared the participants’ age and gender using independent *t*‐tests and chi‐squared tests, respectively. We used repeated‐measure analysis of variance (RM‐ANOVA) to compare MMSE scores between the KGT and placebo groups (i.e., between‐subject effect or BTS effect) before and after treatment conditions (i.e., within‐subject effect or WIS effect), and the group x condition (G x Con) interaction. The post hoc test for pairwise comparisons of subgroups was tested using the Scheffé test. We calculated the absolute difference (abs(Diff) [%]) of MMSE scores using the following formula: I(value before treatment − value after treatment)I × 100% divided by the value before treatment. We compared the abs(Diff) values between the KGT and placebo groups using the independent *t*‐test.

#### 2.4.2 GABA and metabolite data

We tested the values obtained from MEGA PRESS and PRESS SVS for GABA+/Cr, Cho/Cr, NAA/Cr, mI/Cr, and Glx/Cr using RM‐ANOVA to evaluate BTS and WIS effects, and G x Con interaction. The BTS effect was the participant's group effect of the KGT and placebo treatments. The conditions for the WIS effect were the normalized metabolite values before and after treatment. We evaluated the WIS effect for condition and G x Con interaction using Huynh and Feldt^25^ because the estimates for sphericity and epsilon were greater than 0.75. When we found any significant difference, we performed post hoc tests using independent *t*‐tests to compare the KGT or placebo groups and paired *t*‐tests for the before and after treatment conditions. The absolute differences in the abs(Diff) (%) of GABA+/Cr and the normalized metabolite values were calculated and compared between the KGT and placebo groups using independent *t*‐tests.

#### 2.4.3 CBF maps

We performed the statistical analyses for the CBF maps using voxel‐based and region of interest (ROI)–based methods and defined ROI areas based on the voxel‐based analysis. For the voxel‐based analysis, we performed 2 × 2 flexible factorial analysis of CBF maps to define the ROI areas. The three factors were defined as the subject, group, and condition. The subject factor consisted of 30 participants as the main effects, which are always influenced by the number of subjects in each group. The group factor, which consisted of two groups (i.e., 16 and 14 participants in the KGT and placebo groups, respectively), was used to test the BTS effect. The condition factor, which consisted of two conditions (i.e., before and after treatment), was used to test the WIS effect. We evaluated the main effects by groups, conditions, and G x Con interaction. We applied a significance level of *p* = 0.001 without correction for multiple comparisons and clusters with at least 100 contiguous voxels.

For the ROI‐based analysis, we defined the ROI areas using two different methods. First, we defined the cluster‐based ROI areas based on the regions with the most significant difference in the flexible factorial analyses. The cluster‐based ROIs are defined as the clusters at the middle frontal gyrus and the precentral gyrus (Cluster 1) obtained from the result of the two‐group difference, and at the inferior, middle, and superior temporal gyrus (Cluster 2) obtained from the result of the two‐condition difference. Second, the atlas‐based ROIs were defined at the areas of the bilateral hippocampi, the bilateral fusiform gyrus, the bilateral precuneus, and the bilateral posterior cingulate. The values of CBF were extracted from the selected ROIs. We performed RM‐ANOVA to evaluate the effects of these values in the KGT and placebo groups (BTS effect), before and after treatment conditions (WIS effect), and G x Con interaction. If we found any significant difference, then we performed post hoc tests. The abs(Diff) (%) in the CBF values was calculated and each abs(Diff) value between the KGT and placebo groups was compared using the independent *t*‐test. For the ROI analyses, we defined the significance level as a *p*‐value of less than 0.05. We performed the statistical analysis using the Medcalc (MedCalc Software, Acacialaan, Ostend, Belgium) statistical program.

## 3 RESULTS

### 3.1 Demographic data and clinical outcome scores

Table 1 summarizes the statistical results of the demographic data and the neuropsychological tests of the participants with aMCI treated with KGT or placebo medicine. The age (*p* = 0.949) and gender (*p* = 0.352) of the participants in the KGT and placebo groups were not significantly different. However, there were more males than females in both groups. The results of the RM‐ANOVA tests showed that the K‐MMSE scores were significantly different between the KGT and placebo groups (*F* = 4.71, *p* = 0.039), but the results of the post hoc tests showed that the significance disappeared when the group comparisons were performed for the before (*p* = 0.061) and after (*p* = 0.087) treatment conditions separately. K‐MMSE scores were not significantly different between the before and after treatment conditions (*F* = 0.870, *p* = 0.358). K‐MMSE scores did not show G x Con interactions. The absolute difference (abs[Diff] [%]) between the before and after treatment conditions for the MMSE scores was lower in the KGT group than in the place group, but was not significant (*p* = 0.257). Figure S1 shows graphs of the changes in the MMSE scores before and after treatment by KGT and placebo for each participant, and its mean value over the participants is listed in Table 1

### 3.2 GABA and metabolite data

Table 3 summarizes the statistical results of the metabolite and GABA values for each treatment group. The results of the RM‐ANOVA tests showed that the GABA+/Cr values were not significantly different between the before and after treatment conditions (*F* = 0.14, *p* = 0.708). The GABA+/Cr values were significantly different between the KGT and placebo groups (*F* = 5.27, *p* = 0.029). The results of the post hoc tests showed that they were also significantly different between the KGT and placebo groups before treatment (*p* = 0.022), but not after treatment (*p* = 0.224). We also did not find any G x Con interactions for the GABA+/Cr values (*F* = 0.51, *p* = 0.481).

**TABLE 3:** Summary of the metabolite values and the gamma‐aminobutyric acid (GABA) value

	KGT			Placebo			RM‐ANOVA^a^			Group
Metabolite	Before(1a)	After(1b)	abs(Diff)(%)	Before(2a)	After(2b)	abs(Diff)(%)	BTS	WIS	G x Con	abs(Diff) *p*‐value^b^
GABA/Cr	0.08±0.01	0.08±0.01	11.92±8.31	0.09±0.007	0.09±0.01	12.77±9.75	*F* = 5.27 *p* = 0.029 *p* = 0.022 (1a, 2a)^b^	*F* = 0.14 *p* = 0.708	*F* = 0.51 *p* = 0.481	0.798
NAA/Cr	1.56±0.14	1.55±0.10	7.17±5.28	1.60±0.09	1.48±0.34	11.31±17.43	*F* = 0.030 *p* = 0.864	*F* = 2.48 *p* = 0.127	*F* = 1.56 *p* = 0.223	0.373
Cho/Cr	0.68±0.06	0.66±0.06	6.89±5.36	0.70±0.04	0.69±0.05	5.81±4.17	*F* = 1.98 *p* = 0.171	*F* = 2.03 *p* = 0.165	*F* = 0.86 *p* = 0.362	0.549
mI/Cr	0.74±0.30	0.69±0.11	19.25±18.39	0.65±0.12	0.70±0.12	16.84±13.75	*F* = 0.52 *p* = 0.478	*F* = 0.0023 *p* = 0.962	*F* = 1.39 *p* = 0.249	0.691
Glx/Cr	1.96±0.42	1.79±0.37	15.27±9.47	1.98±0.35	2.30±0.70	34.59±40.93	*F* = 4.19 *p* = 0.050	*F* = 0.41 *p* = 0.529	*F* = 4.21 *p* = 0.050	0.077

*Note*: The data are presented as the mean ± standard deviation. This table summarizes the results of the metabolite values from single‐voxel Point‐RESolved Spectroscopy (PRESS) and the GABA value from Meshcher–Garwood PRESS normalized by the creatine level obtained in the participants with amnestic mild cognitive impairment before and after treatments with KGT or placebo medicine. The abs(Diff) (%) is the absolute difference of each value between before and after treatments, divided by the value before treatment, and then multiplied by 100%. The abs(Diff) value was compared between the KGT and placebo groups by using the independent *t*‐test.

Abbreviations: BTS, between‐subject group; Cho/Cr, choline (Cho) divided by total creatine (Cr); GABA/Cr, gamma‐aminobutyric acid (GABA) divided by Cr; Glx/Cr, glutamate complex (Glx) divided by Cr (sum of 2.11 ppm through 2.45 ppm); KGT, Kami Guibi‐tang; mI/Cr, myo‐Inositol divided by Cr (sum of 3.55 ppm through 3.63 ppm); NAA/Cr, N‐acetylaspartate (NAA) divided by Cr; RM‐ANOVA, repeated‐measure analysis of variance; WIS, within‐subject condition.

^a^ Result by RM‐ANOVA: The between‐subject effect was the participant's group of the 16 KGT and 14 placebo treatments. The condition for the within‐subject factor was GABA/Cr and metabolite values before and after treatment. The test of within‐subject effect was evaluated for condition and group x condition interaction. G x Con is the group x condition interaction using Huynh–Feldt. The degree of freedom was 1.

^b^ Result for independent sample *t*‐test.

The metabolite values for NAA/Cr, Cho/Cr, mI/Cr, and Glx/Cr were not significantly different between the before and after treatment conditions. Furthermore, those values did not have any group x condition (G x Con) interactions. The abs(Diff [%]) in the Glx/Cr values between the before and after treatment conditions was lower in the KGT group than in the placebo groups, but was not statistically significant (*p* = 0.077). Figure S2 shows the graphs of the changes in the metabolites and GABA before and after treatment by KGT and placebo for each participant, and its mean value over the participants is listed in Table 3

### 3.3 CBF maps

#### 3.3.1 Voxel‐based result

**FIGURE 3:**
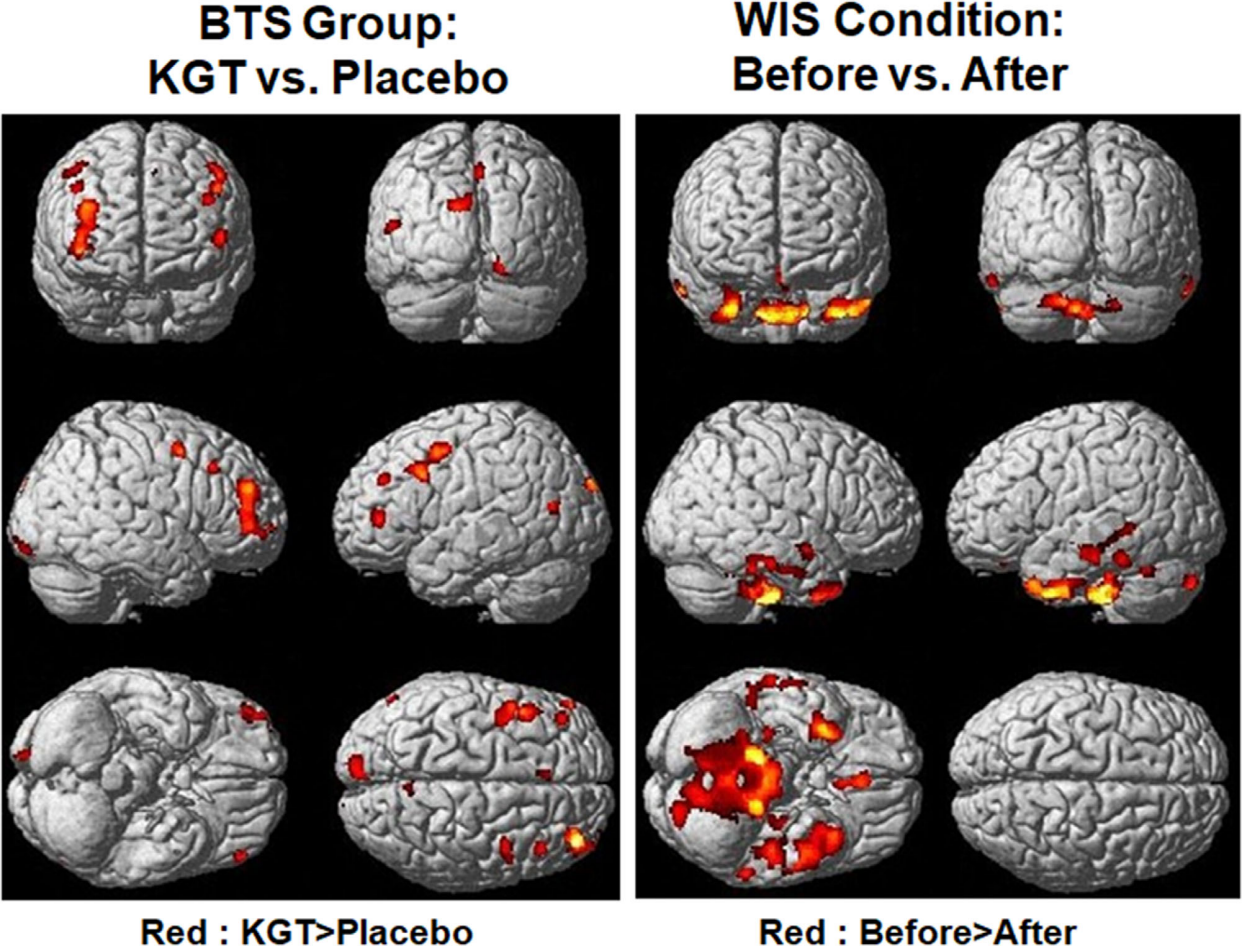
Results of voxel‐based flexible factorial analyses of cerebral blood flow (CBF) maps; the group factor was 2 groups (i.e, 16 for Kami Guibi‐tang and 14 for placebo) for the between‐subject effect. The condition factor was two conditions (i.e., before and after treatment) for the within‐subject effect. There was no group x condition interaction for gray matter volume (GMV), white matter volume (WMV), and CBF maps. We applied a significance level of *p* = 0.001 without correction for multiple comparisons and clusters with at least 100 contiguous voxels. The voxel‐based analysis was performed to define the region‐of‐interest areas

Figure 3 shows the results of the voxel‐based flexible factorial analyses of the CBF maps. The CBF values were significantly different between before and after treatment conditions and were higher before treatment than after treatment at the left inferior and superior and middle temporal gyrus. This cluster was defined as an ROI (Cluster 2). Other significantly different areas were at the left medial frontal gyrus and right inferior and superior temporal gyrus. However, the post hoc tests did not show any significant difference between the two treatment conditions for both the KGT and placebo groups. We did not find any G x Con interactions for the CBF values. The CBF values were significantly different between the KGT and placebo groups. They were higher in the KGT group than in the placebo group at the left middle frontal gyrus and the left precentral gyrus. This cluster was defined as an ROI (Cluster 1). Other significantly different areas were at the left inferior frontal gyrus, left middle occipital gyrus, right middle frontal gyrus, and right precentral gyrus. However, the post hoc tests showed that this significant difference disappeared for both before and after treatment conditions.

#### 3.3.2 ROI‐based result

The results of the ROI‐based analyses are summarized in Table 4 for the CBF. We found a significant difference between the two treatment conditions. The RM‐ANOVA results showed that the CBF values were significantly lower after treatment at Cluster 2 for the KGT group (*p* = 0.003) and the placebo group (*p* = 0.011), at the hippocampus for the KGT group (*p* = 0.004) and the placebo group (*p* = 0.008), and at the fusiform gyrus for the KGT group (*p* = 0.002). Furthermore, the CBF values were significantly lower in the placebo group than in the KGT group both before (*p* = 0.019) and after (*p* = 0.005) the treatment. The abs(Diff [%]) of the CBF values between the before and after treatment conditions at the fusiform gyrus was significantly lower in the KGT group than in the placebo groups (*p* = 0.024).

**TABLE 4:** Summary of the CBF in each area

	KGT			Placebo			RM‐ANOVA^a^			Group
ROI	Before (1a)	After (1b)	abs(Diff.)(%)	Before (2a)	After (2b)	abs(Diff.)(%)	BTS	WIS	G x Con	abs(Diff) *p*‐value^b^
Cluster 1	109.10±42.55	113.05±44.20	25.22±17.67	72.10±35.42	70.47±30.76	26.14±9.44	*F* = 8.71 *p* = 0.006 *p* = 0.019 (1a, 2a)^b^ *p* = 0.005 (1b, 2b)^b^	*F* = 0.017 *p* = 0.898	*F* = 0.41 *p* = 0.526	0.864
Cluster 2	56.77±38.18	31.34±24.12	58.75±44.98	61.0830.65	26.85±24.42	79.11±43.97	*F* < 0.001 *p* = 0.992	*F* = 20.07 *p* < 0.001 *p* = 0.003 (1a, 1b)^c^ *p* = 0.011 (2a, 2b)^c^	*F* = 0.44 *p* = 0.514	0.222
Hippo	83.85±26.51	72.59±25.92	18.65±8.84	85.22±23.03	71.19±17.88	21.01±10.41	*F* < 0.001 *p* = 0.999	*F* = 21.42 *p* < 0.001 *p* = 0.004 (1a, 1b)^c^ *p* = 0.008 (2a, 2b)^c^	*F* = 0.26 *p* = 0.616	0.507
Fusiform	70.00±27.11	58.47±21.43	16.63±11.40	63.20±19.26	55.83±16.44	28.91±16.62	*F* = 0.41 *p* = 0.526	*F* = 10.27 *p* = 0.003 *p* = 0.002 (1a, 1b)^c^	*F* = 0.50 *p* = 0.485	0.024
Precuneus	62.95±21.85	63.69±27.73	24.65±19.34	59.07±25.29	58.31±18.88	25.28±19.28	*F* = 0.19 *p* = 0.666	*F* < 0.086 *p* = 0.771	*F* = 0.38 *p* = 0.543	0.930
Posterior Cingulate	84.81±25.21	78.51±26.68	17.66±8.79	85.22±23.27	74.06±17.94	20.27±9.93	*F* = 0.063 *p* = 0.804	*F* = 7.36 *p* = 0.011 *p* = 0.039 (2a, 2b)^c^	*F* = 0.57 *p* = 0.457	0.450

*Note*: The data are presented as the mean ± standard deviation. The abs(Diff) (%) is the absolute difference of the CBF value between before and after treatments, divided by the value before treatment, and then multiplied by 100% for each ROI. The abs(Diff) value was compared between the KGT and placebo groups by using the independent *t*‐test. The cluster‐based ROI are defined as the clusters at the middle frontal gyrus and the precentral gyrus (Cluster 1) obtained from the result of the two‐group difference and at the inferior, middle, and superior temporal gyrus (Cluster 2) obtained from the result of the two‐condition difference. In addition, the atlas‐based ROIs are defined at the areas of the bilateral hippocampi (Hippo), the bilateral fusiform gyrus (Fusiform), the bilateral precuneus, and the bilateral posterior cingulate.

Abbreviations: BTS, between‐subject group; CBF, cerebral blood flow; KGT, Kami Guibi‐tang; RM‐ANOVA, repeated‐measure analysis of variance; ROI, region of interest; WIS, within‐subject condition.

^a^ Result for RM‐ANOVA: The between‐subject effect was the participant's group of the 16 KGT and 14 placebo treatments. The condition for the within‐subject factor was CBF before and after treatment. The test of within‐subject effect was evaluated for condition and group x condition interaction. G x Con is the group x condition interaction using Huynh–Feldt. The degree of freedom was 1.

^b^ Result for independent sample *t*‐test.

^c^ Result for paired *t*‐test.

The RM‐ANOVA results showed that the CBFs at only Cluster 1 were significantly different between the KGT and placebo groups (*F* = 8.71, *p* = 0.006). The post hoc tests showed that the CBFs were significantly lower in the placebo group than in the KGT group both before (*p* = 0.019) and after (*p* = 0.005) the treatment. We did not find any G x Con interactions for all the ROIs. Figure S3 shows the graphs of the changes of the CBF before and after treatment by KGT and placebo for each participant and for each brain area, and its mean value over the participants is listed in Table 4.

## 4 DISCUSSION

### 4.1 Impact of KGT on memory improvement

Several reports have shown that KGT improves memory function. A previous study reported that KGT improved the memory deficit of AD mice by affecting their cortical axons and presynaptic terminals.^8^ Therefore, KGT may increase the efficiency of synapses rather than induce changes in the GM and WM. Another study showed that KGT inhibited tau phosphorylation and restored atrophied axons in the AD model.^26^ In this study, we used MRI measures of metabolites, GABA levels, and CBF to evaluate the effect of KGT on brain function in patients with aMCI. The long‐term measurement of these MRI indices may be needed to effectively evaluate the improvement of axonal functions.

### 4.2 Metabolites and GABA measures

The metabolites and GABA levels were not significantly different before and after the KGT treatment. We expected to measure significant effects for GABA and brain metabolites with KGT because previous studies showed that a greater Glx concentration was correlated with a higher memory performance.^27^, ^28^, ^29^ A previous study also showed that the Glx/Cr value decreased in the drug treatment group with schizophrenia.^30^ The reduction of Glx/Cr after treatment can be related to improvement of the inhibitor of the glutamatergic system. Additionally, the improvement of the NAA concentration is also a marker for improved neuronal function in AD^31^ and MCI.^32^ We can explain our results by the following two standpoints: first, GABA or brain metabolites were not significantly changed after taking KGT because of the location of the voxel of interests (VOIs), which were at the precuneus and posterior cingulate areas and maybe not directly affected by the KGT treatment. The precuneus is a region with various functions, including episodic memory retrieval,^33^ and the medial temporal gyrus is involved in cognitive processes, such as language and semantic memory processing.^34^ Therefore, the middle temporal gyrus areas, the middle frontal gyrus areas, and the hippocampus should be the candidates for location of the VOIs. Second, a KGT treatment of 24 weeks was not enough to have a significant impact on the level of GABA or brain metabolites in aMCI patients. Additionally, although the menstrual cycle may affect GABA in female subjects,^35^ we do not think that it would have contributed to the outcome because this study was conducted with elderly participants.

### 4.3 Reduced CBF after KGT treatment

The ROI‐based results showed that the CBF values were significantly different before and after treatments. The CBF values were reduced after 24 weeks of medication in both groups. We showed that there were lower CBF values in the KGT and placebo groups after treatment, but the abs(Diff [%]) values were significantly different between the two treatment groups at the fusiform gyrus (*p* = 0.024). A previous study showed drug‐induced CBF decrease in the fusiform area after a single oral dose of citalopram.^36^ Although the fusiform gyrus is known to be involved in face recognition, it also has been showed to interact with the amygdala, which functions to modulate emotional stimuli.^37^ It is possible that CBF of the fusiform gyrus decreased because KGT reduced depression or emotional stress according to the effect of the drug.

The lower CBF after treatment may be due to a compensatory response to maintain the memory function. Previous studies have showed that the CBF value was altered to change the memory function. For example, a higher CBF in the hippocampus and the posterior cingulate was related to poorer memory performance in amyloid‐β positive older adults.^38^ Another study showed a higher CBF value in MCI patients than in control.^39^ Because K‐MMSE scores were also not significantly different between the treatment conditions, we did not expect an association between CBF values and MMSE scores.^38^ These results can first be explained by the low number of participants in each group, which can be responsible for the nonsignificance of the statistical results. In future studies, we would need to analyze a relatively large population. Second, our KGT treatment regimen may be too weak to show any effect on CBF in aMCI patients. Therefore, we would recommend extending the duration of the KGT treatment in a future study.

### 4.4 Limitations of this study

This is the first neuroimaging study observing the effect of KGT in aMCI patients that was effective in measuring the improvement of cognitive function compared to other clinical and basic studies. However, our study has several limitations First, AD is more frequent in women than in men. However, we enrolled more men than women in each group. In future studies, we should enroll more women than men. Second, the number of participants in each group was small and therefore future studies should be performed with a large population. Third, this current study was performed in aMCI participants. It may be necessary to study AD patients who experience more severe cognitive impairments or to extend the duration of the KGT treatment. Finally, further studies with longer KGT treatments are needed to compare the treatment effect based on clinical observations and MRIs.

## 5 CONCLUSION

In this study, we aimed to assess the effects of KGT in aMCI patients by quantifying the changes in brain metabolites and CBFs. The CBF measure showed the treatment effect of KGT on MCI participants. We found that the treatment affected the temporal lobe, including the hippocampus and the fusiform gyrus, which are associated with memory. On the other hand, we measured almost no significant changes in brain metabolites, which may be related to not enough dosage of KGT to improve the cognitive values. Therefore, further studies should be performed with a relatively larger population and increased dosage.

## CONFLICT OF INTEREST

The authors declare that they have no conflict of interest.

## AUTHOR CONTRIBUTIONS

Seun‐Yeon Cho and Sharonkyuhee Kwon: writing – original draft. Hee‐Yeon Shin, Ha‐Ri Kim, and Jeong‐Hwa Kim: investigation. Soonchan Park and Chang‐Woo Ryu: writing – review and editing. Jung‐Mi Park: conceptualization; writing – review and editing, project administration, supervision, and funding acquisition. Richard Edden: software. Geon‐Ho Jahng: formal analysis; writing – original draft, supervision, and funding acquisition.

## Supporting information



Supporting Information

## Data Availability

The datasets generated and analyzed during the current study are available from the corresponding author on reasonable request.
