# Supplementary material for: Treatment evaluation of Kami Guibi‐tang on participants with amnestic mild cognitive impairment using magnetic resonance imaging on brain metabolites, gamma‐aminobutyric acid, and cerebral blood flow
Source: J Appl Clin Med Phys. 2021 Oct 11;22(11):151–64. doi: 10.1002/acm2.13443 (PMC8598148; doi:10.1002/acm2.13443)
Supplement: Supplementary file 1 — Supporting Information [file ACM2-22-151-s001.docx]

**Supplementary Figure**


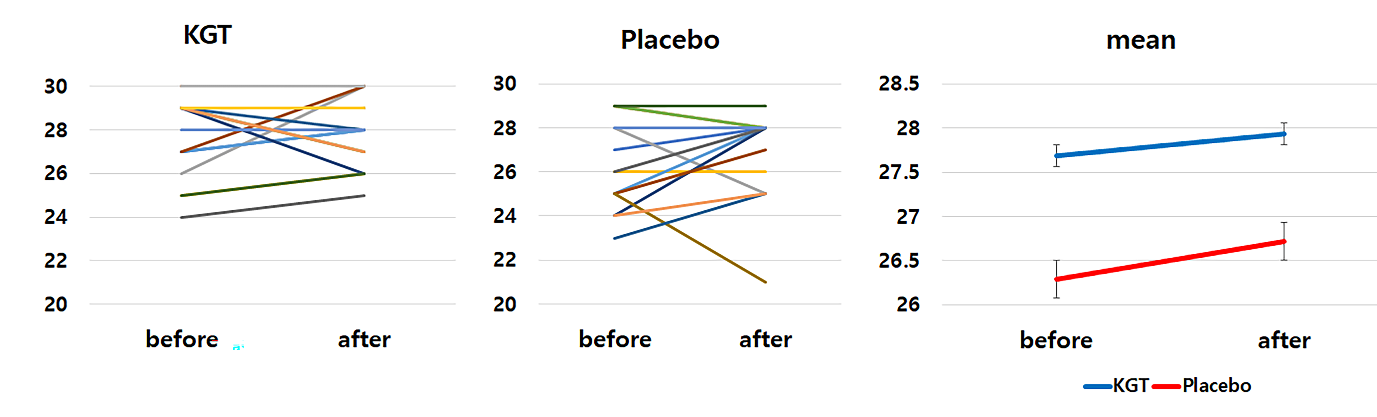


**Supplementary Figure S1**. Graphs of the changes of the Mini-Mental State Examination (MMSE) scores before and after treatment by KGT and placebo for each participant and its mean value over the participants listed in **Table 1**.

The vertical line in mean indicates standard deviation over the participant group.


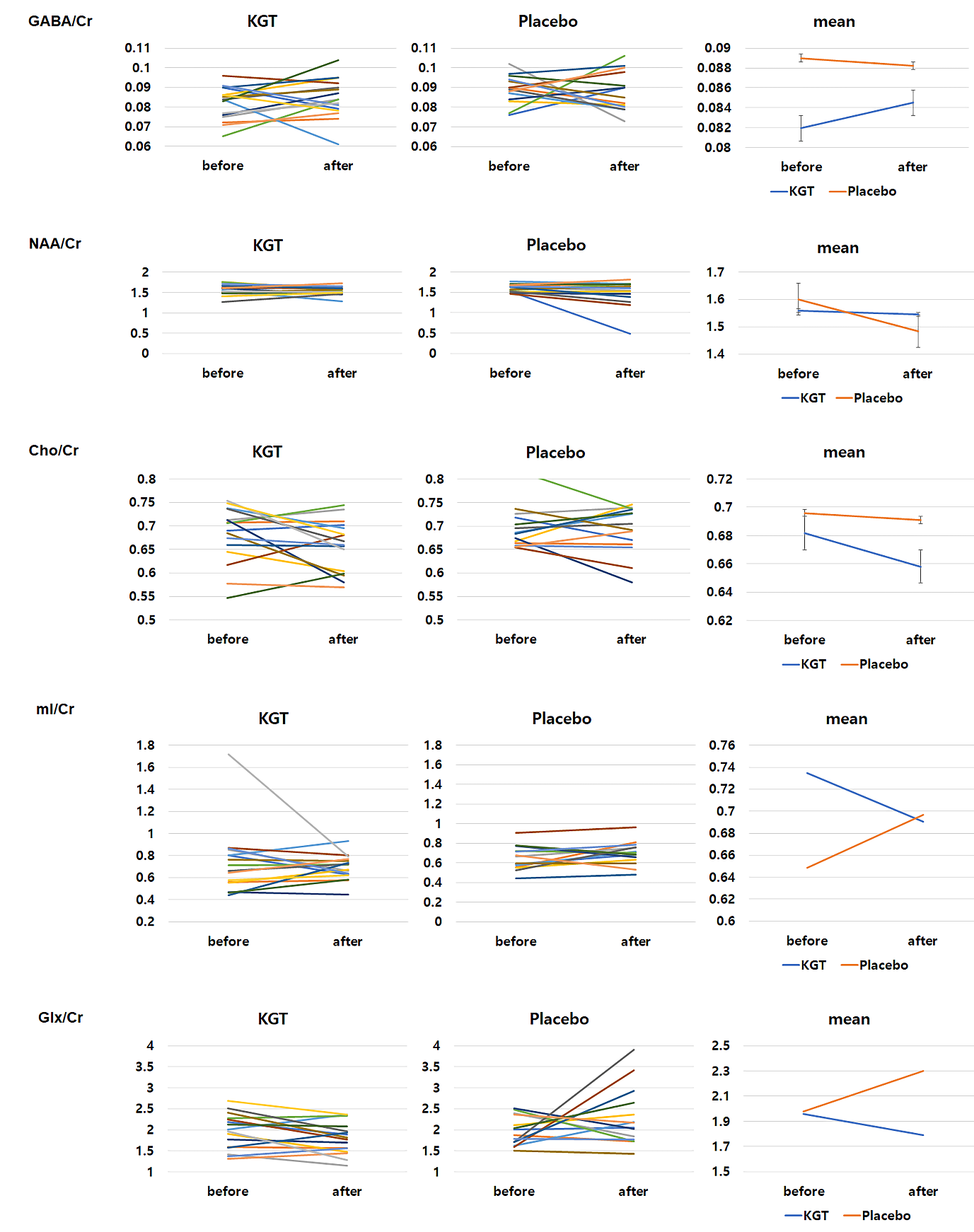


**Supplementary Figure S2**. Graphs of the changes of the metabolites and gamma-aminobutyric acid (GABA) before and after treatment by KGT and placebo for each participant and its mean value over the participants listed in **Table 3**.

The vertical line in mean indicates standard deviation over the participant group.


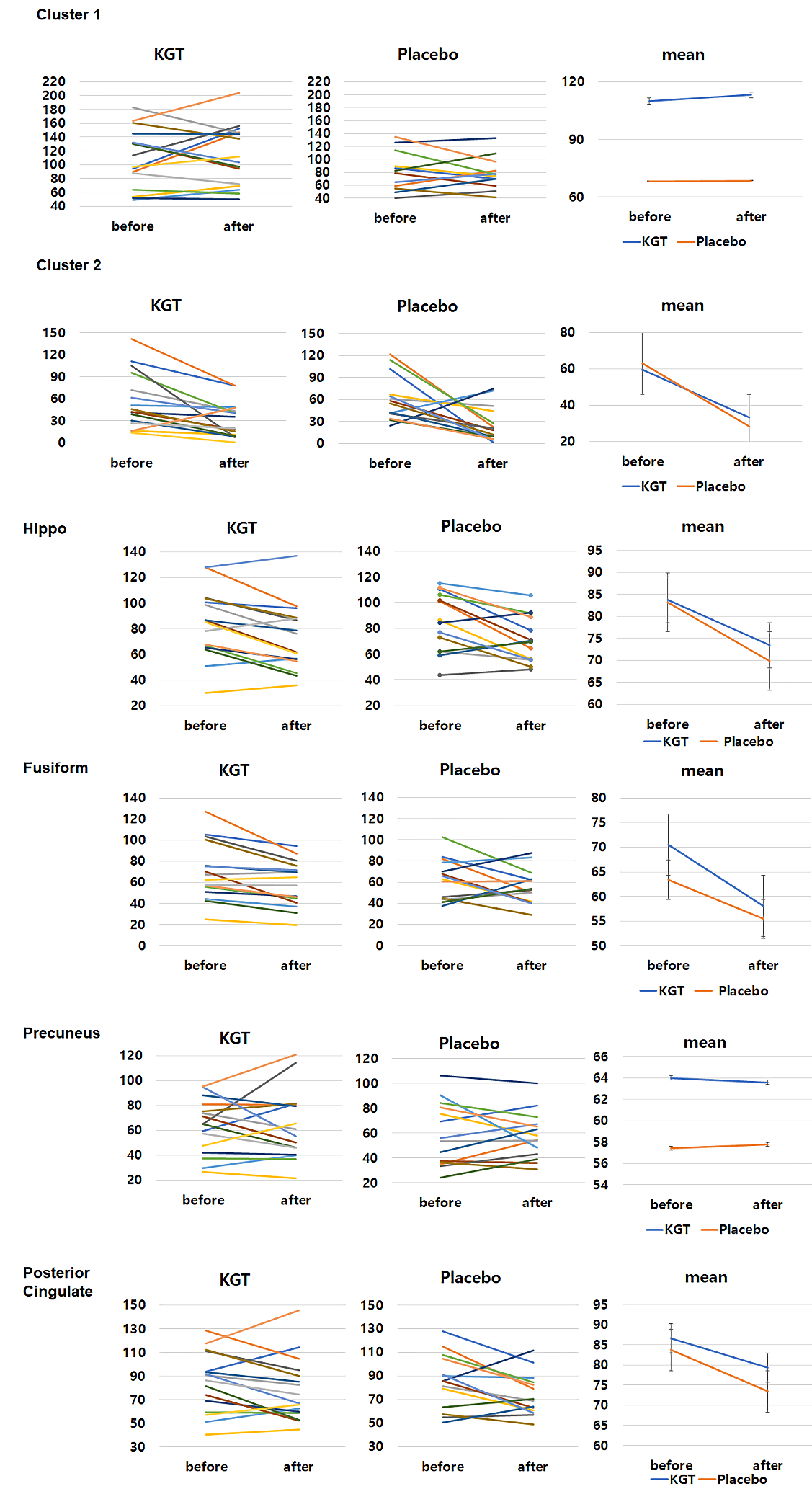


**Supplementary Figure S3**. Graphs of the changes of the cerebral blood flow (CBF) before and after treatment by KGT and placebo for each participant and for each brain area and its mean value over the participants listed in **Table 4**.

The vertical line in mean indicates standard deviation over the participant group.
